# Inter-study repeatability of circumferential strain and diastolic strain rate by CMR tagging, feature tracking and tissue tracking in ST-segment elevation myocardial infarction

**DOI:** 10.1007/s10554-020-01806-8

**Published:** 2020-03-09

**Authors:** Sheraz A. Nazir, Abhishek M. Shetye, Jamal N. Khan, Anvesha Singh, Jayanth R. Arnold, Iain Squire, Gerry P. McCann

**Affiliations:** Department of Cardiovascular Sciences, University of Leicester and the National Institute for Health for Research (NIHR) Leicester Cardiovascular Biomedical Research Centre, Glenfield Hospital, Leicester, LE3 9QF UK

**Keywords:** Repeatability, Tagging, Feature tracking, Tissue tracking, STEMI

## Abstract

Strain assessment allows accurate evaluation of myocardial function and mechanics in ST-segment elevation myocardial infarction (STEMI). Strain using cardiovascular magnetic resonance (CMR) has traditionally been assessed with *tagging* but limitations of this technique have led to more widespread use of alternative methods, which may be more robust. We compared the inter-study repeatability of circumferential global peak-systolic strain (*Ecc*) and peak-early diastolic strain rate (PEDSR) derived by tagging with values obtained using novel cine-based software: Feature Tracking (FT) (TomTec, Germany) and Tissue Tracking (TT) (Circle cvi^42^, Canada) in patients following STEMI. Twenty male patients (mean age 56 ± 10 years, mean infarct size 13.7 ± 7.1% of left ventricular mass) were randomised to undergo CMR 1–5 days post-STEMI at 1.5 T or 3.0 T, repeated after ten minutes at the same field strength. *Ecc* and PEDSR were assessed using tagging, FT and TT. Inter-study repeatability was evaluated using Bland–Altman analyses, coefficients of variation (CoV) and intra-class correlation coefficient (ICC). *Ecc* (%) was significantly lower with tagging than with FT or TT at 1.5 T (− 9.5 ± 3.3 vs. − 17.5 ± 3.8 vs. −15.5 ± 5.2, respectively, p < 0.001) and 3.0 T (− 13.1 ± 1.8 vs. − 19.4 ± 2.9 vs. − 17.3 ± 2.1, respectively, p = 0.001). This was similar for PEDSR (.s^−1^): 1.5 T (0.6 ± 0.2 vs. 1.5 ± 0.4 vs. 1.0 ± 0.4, for tagging, FT and TT respectively, p < 0.001) and 3.0 T (0.6 ± 0.2 vs. 1.5 ± 0.3 vs. 0.9 ± 0.3, respectively, p < 0.001). Inter-study repeatability for *Ecc* at 1.5 T was good for tagging and excellent for FT and TT: CoV 16.7%, 6.38%, and 8.65%, respectively. Repeatability for *Ecc* at 3.0 T was good for all three techniques: CoV 14.4%, 11.2%, and 13.0%, respectively. However, repeatability of PEDSR was generally lower than that for *Ecc* at 1.5 T (CoV 15.1%, 13.1%, and 34.0% for tagging, FT and TT, respectively) and 3.0 T (CoV 23.0%, 18.6%, and 26.2%, respectively). Following STEMI, *Ecc* and PEDSR are higher when measured with FT and TT than with tagging. Inter-study repeatability of *Ecc* is good for tagging, excellent for FT and TT at 1.5 T, and good for all three methods at 3.0 T. The repeatability of PEDSR is good to moderate at 1.5 T and moderate at 3.0 T. Cine-based methods to assess *Ecc* following STEMI may be preferable to tagging.

**Electronic supplementary material:**

The online version of this article (10.1007/s10554-020-01806-8) contains supplementary material, which is available to authorized users.

## Background

ST-segment elevation myocardial infarction (STEMI) is associated with left ventricular (LV) systolic and diastolic dysfunction [1, 2]. Myocardial strain (defined as the change in length of an object relative to its original length) is a sensitive measure of contractility, which can be calculated in a variety of coordinate systems at both the segmental and global level and it is typically determined in the three axes of myocardial contraction—circumferential, longitudinal and radial [3]. Strain rate measures the change in strain for a given vector as a function of time. Global myocardial circumferential peak-systolic strain (*Ecc*) and peak-early diastolic strain rate (PEDSR) are objective, sensitive markers of myocardial systolic and diastolic function [1, 4]. In STEMI, both global longitudinal strain (GLS) and *Ecc* determined by speckle-tracking echocardiography independently predict adverse LV remodelling and prognosis, however, circumferential strain rate may be a more powerful predictor of long-term adverse LV remodelling [1, 5, 6]. Cardiovascular magnetic resonance (CMR) offers superior tissue contrast, spatial resolution and signal-to-noise-ratio compared with echocardiography [7]. Additionally, CMR is the gold-standard technique for LV volumetric assessment and infarct size (IS) quantification [8, 9]. *Ecc* detected by CMR can predict functional recovery, worse long-term outcomes and provide additional prognostic information beyond conventional clinical and CMR variables (not shown for GLS [10]) in patients with a first STEMI [11, 12]. Furthermore, *Ecc* maybe the most reproducible strain parameter with the least inter-technique variation [13–17]. PEDSR is a sensitive marker of diastolic dysfunction that may occur early in STEMI, independent of systolic dysfunction, which is associated with adverse outcomes [2].

Strain on CMR has traditionally been assessed using tissue tagging (saturated perpendicular tag lines applied to myocardial tissue to track cardiac motion) [7]. However, its clinical utility is hampered by the need to acquire additional sequences, time-consuming post-processing and diastolic tag fading [18, 19]. Feature tracking (FT) (TomTec, Germany) and Tissue Tracking (TT) (Circle cvi^42^, Canada) assess strain on routinely acquired balanced steady-state free precession (SSFP) cine sequences. FT follows distinctive characteristics at endocardial- and epicardial-cavity borders, akin to speckle tracking, to track myocardial deformation [20]. TT uses a ‘mid-surface curvilinear coordinate system’ to track LV deformation and follows the motion of software-generated myocardial nodes on SSFP cine sequences through the cardiac cycle to compute strain [21]. However, an advantage with TT is that volumetric, functional and strain analysis can be performed on a single software platform, without re-contouring per sequence. Strain assessed by FT and TT has been shown to predict major adverse cardiovascular events following STEMI [11, 22].

Previous studies have assessed inter- and intra-observer variability of tagging in various study populations [18, 23, 24]. Additionally, observer variability of FT at both 1.5 T and 3.0 T CMR has been shown to be similar to that of tagging although, in STEMI patients, it may be better with FT [14, 18, 24]. Whilst inter-study repeatability, a key determinant to power interventional studies, has been assessed for FT and TT previously in a variety of populations [25, 26], no study has evaluated this for all three platforms in the STEMI population at both clinically utilised magnetic field strengths.

This study aimed to assess the inter-study repeatability of *Ecc* and PEDSR evaluated by tagging, FT and TT at 1.5 T and 3.0 T in patients with STEMI.

## Methods

### Study population and recruitment

Twenty patients presenting to a single, regional cardiac centre with STEMI between November 2014 and April 2015 were prospectively recruited. Inclusion criteria included male gender, age ≥ 18 years and a definitive diagnosis of STEMI (based on European Society of Cardiology guidelines [27]) reperfused by primary percutaneous coronary intervention. Patients with systolic blood pressure ≤ 90 mmHg, cardiogenic shock, stage 4/5 kidney disease (estimated Glomerular Filtration Rate < 30 ml^−1^ min^−1^ 1.73^2^) and contraindications to CMR were excluded. The United Kingdom National Research Ethics Service approved the study (14/LO/1917) and all patients provided their written informed consent prior to their inclusion in the study.

### Study design

Patients were randomised 1:1, using the software MinimPy Program 0.3 © (distributed under GNU General Public License version 3.0) [28], to undergo CMR either at 1.5 T (Siemens Avanto, Erlangen, Germany) or 3.0 T (Siemens Skyra, Erlangen, Germany). Allocation was stratified by infarct location (anterior/non-anterior) and automatically performed by the MinimPy randomisation software since infarct location was entered as a pre-defined stratification variable during the setup phase [28, 29].

### CMR image acquisition

**Fig. 1:**
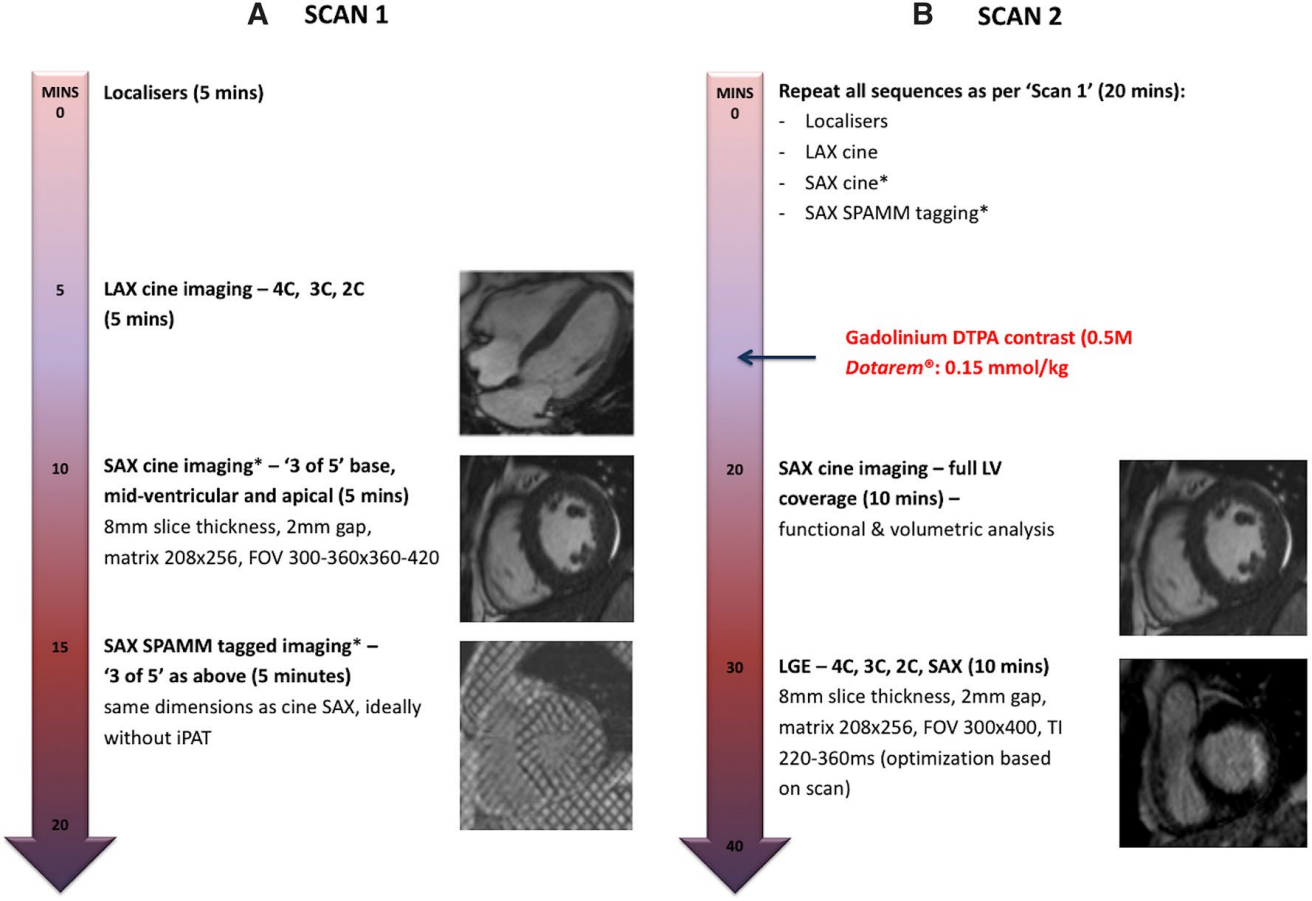
CMR Imaging Protocol. Abbreviations: *SPAMM* spatial modulation of magnetisation; *FOV* field of view; *iPAT* integrated parallel acquisition technique, *LAX* long axis, *LGE* late gadolinium enhancement, *LV* left ventricle, *SAX* short axis, *TI* inversion time. *Order of SAX cine and tagging acquisitions reversed between patients

The imaging protocol is presented in Fig. 1. Specifically, breath-held and retrospectively ECG gated SSFP images were acquired using either a 1.5 T (using a 6-channel phased array cardiac coil) or 3.0 T (using an 18-channel cardiac coil) platform to cover the entire left ventricle. Typical imaging parameters were: 8 mm slice thickness with 2 mm gap, matrix size 208 × 256, field of view 300–360 × 360–420 mm, temporal resolution ~ 48 ms, echo time (TE) 1.21 ms, with 30 reconstructed phases.

#### First scan

Cine SSFP sequences were acquired in long-axis (2/3/4-chamber) views and in three (basal, mid-ventricular and apical) short-axis (SAX) locations. Tagged images were acquired in identical SAX positions using a prospectively gated spatial modulation of magnetization (SPAMM) gradient-echo sequence as previously described [18]. The order in which tagging and cine SSFP sequences were performed was random ensuring that chronology of sequence acquisition and potential associated breath-holding fatigue did not bias results.

#### Second scan

The patient was removed from the scanner following the first scan, re-positioned and re-scanned after ten minutes. Three SAX SPAMM-tagging images and three SSFP cine SAX slices were again acquired at identical base, mid-cavity and apical levels. The order in which tagging and cine SSFP sequences were performed was reversed from the first scan to further limit potential bias from possible breath-holding fatigue. A contiguous cine SSFP SAX stack covering the whole left ventricle was acquired immediately following administration of 0.15 mmol/kg of gadoterate meglumine (Dotarem, Guebert S.A., Villepinte, France) for mass and volumetric analysis. Late gadolinium enhancement (LGE) imaging was acquired ten minutes following contrast administration in long axis (2/3/4-chamber) views and contiguous SAX slices covering the whole left ventricle, using a segmented inversion-recovery gradient-echo sequence with progressive adjustment of the inversion time to null unaffected myocardium.

### CMR image analysis

All images were analysed offline on dedicated workstations by 2 readers (SN and AS) blinded to all patient details and scan chronology. Each reader analysed the first and second scans for the same patient.

#### Quantification of LV volumes and infarct size

Volumetric analysis and IS quantification were undertaken using *cvi42* (Circle Cardiovascular Imaging, Calgary, Canada). LV volumes, mass and function were calculated as previously described [30]. IS was quantified on LGE images using the Full-Width Half-Maximum technique [31] and expressed as a percentage of LV mass.

#### Strain analysis

Segmental *Ecc* and PEDSR were quantified using tagging, FT and TT, based on the 16-segment model using the exact same location positions of basal, mid-ventricular and apical myocardium on SAX images for all three techniques, for both the first and second scans [32]. Segmentation was performed by demarcation of the right ventricular insertion point at all three slices for all three methods. Global values of *Ecc* and PEDSR were calculated as an average of the sixteen segments. *Ecc* was defined as the most *negative* strain value (since there is shortening of the circumferential LV myocardial fibres during systole) whilst PEDSR was defined as the most *positive* strain rate value (due to lengthening of the circumferential fibres) during early diastole (usually between 270 and 530 ms of cardiac cycle).

##### *Tagging*

**Fig. 2:**
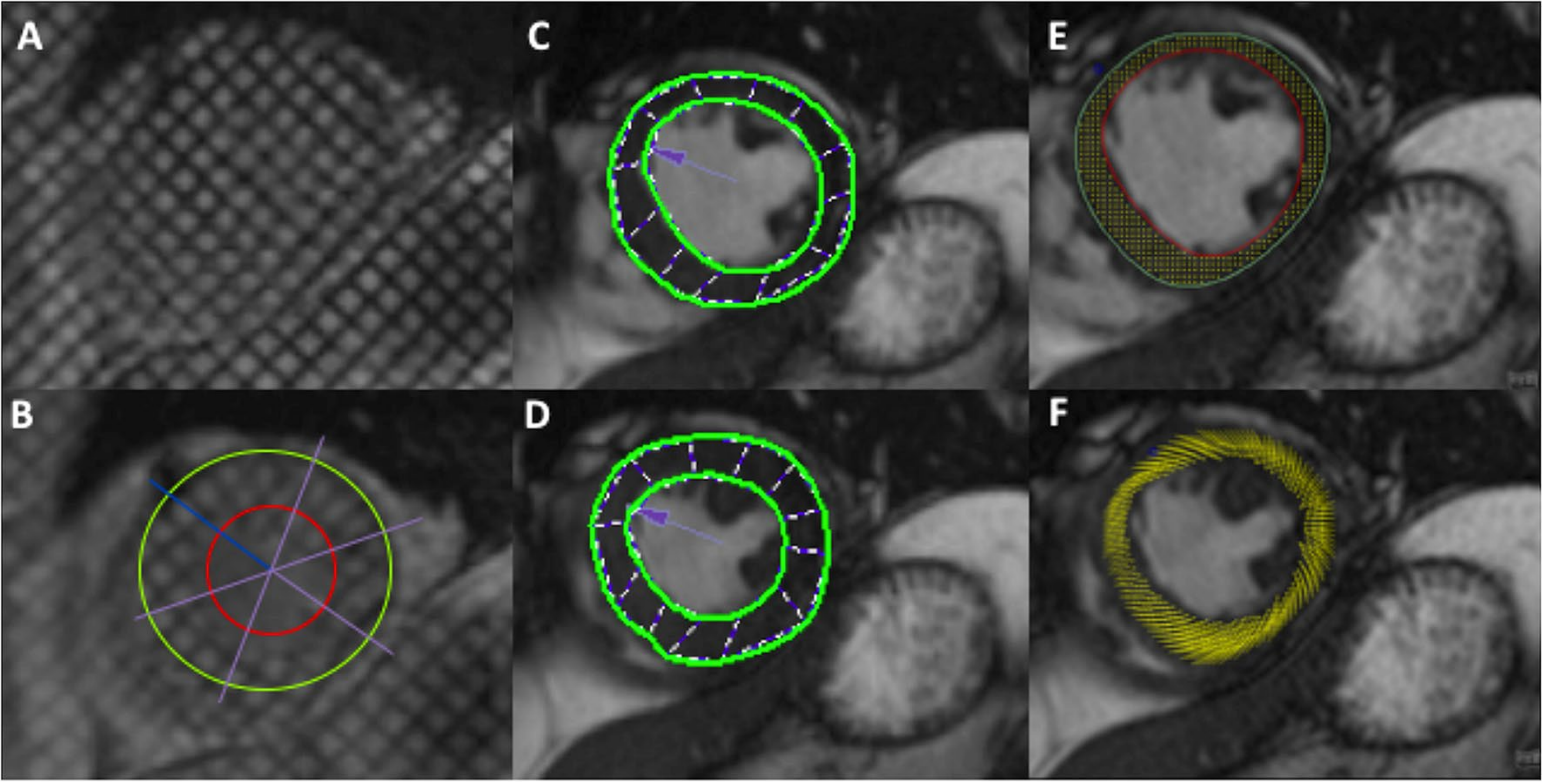
Global strain assessment by tagging (**a** & **b**), Feature Tracking (**c** & **d**) and Tissue Tracking (**e** & **f**). Notes: *End-Diastole* depicted in **a**, **c** and **e**; *End-Systole* depicted in **b**, **d** and **f**. All images are mid-ventricular short axis (SAX) slice

Tagging analysis was performed using the local sine-wave modelling algorithm within the *InTag* post-processing plugin (Creatis, Lyon, France) for *OsiriX* v6.5 (Pixmeo, Switzerland) as previously described (see Fig. 2) [33]. Segmental values of *Ecc* and PEDSR were then further processed using in-house Microsoft Excel v2010 (California, USA) software.

##### *Feature tracking*

FT analysis was performed on Diogenes Image Arena v6.3 (Tomtec, Munich, Germany) software as previously described (see Fig. 2) [20]. Briefly, endocardial and epicardial borders were manually defined at end-diastole on cine images and propagated throughout the cardiac cycle. Contours were manually adjusted where tracking was sub-optimal. The software generated separate *Ecc* and PEDSR values for epicardial and endocardial contours, which were averaged to give a mean value of *Ecc* and PEDSR for each segment. Notably, FT software automatically denotes dyskinetic segments as having a strain value of zero; consequently, all segments had negative *Ecc* and positive PEDSR values.

##### *Tissue tracking*

TT analysis was performed using the TT plug-in for *cvi42* (Circle Cardiovascular Imaging, Calgary, Canada [21]) on the three cine SSFP SAX slices (Fig. 2). End-diastolic endocardial and epicardial contours were propagated with manual re-adjustments performed as required. LV extent was defined on the long axis 4-chamber slice to ensure the software recognised the three SAX slices as equidistant. Global *Ecc* and PEDSR values were automatically generated.

### Statistical analysis

Normality was assessed using the Shapiro–Wilk test, histograms and Q–Q plots. Normally distributed data are shown as mean ± SD and non-parametric data as median (25–75% quartiles). Differences between the 1.5 T and 3.0 T CMR cohorts were assessed using independent t-test (for continuous data) or Fisher’s exact test (for categorical data). Data generated by the three strain analysis methods were compared using analysis of variance (ANOVA) of repeated measures [34]. Pairwise comparison was performed using paired t-tests for absolute values of *Ecc* and PEDSR. Correlation of *Ecc* and PEDSR with total IS, using the values obtained on all 20 scans at both field strengths, was assessed using Pearson’s correlation coefficient (r). Inter-study repeatability was assessed using the Bland–Altman method [35], coefficient of variation (CoV) and two-way mixed-effect intra-class correlation coefficient for absolute agreement [36, 37]. Additionally, sample sizes required to detect a 10% relative change in *Ecc* and PEDSR derived using tagging, FT and TT, with a power of 90% and an alpha error of 0.05, were calculated [38]. Statistical analyses were performed using *GraphPad Prism version 7.0e for Mac OS X* (GraphPad Software, La Jolla California USA, www.graphpad.com) and *Statistical Package for Social Sciences, SPSS version 22.0* (Chicago, IL, USA). A p-value of < 0.05 was considered statistically significant.

## Results

### Population characteristics

Patient characteristics are listed in Table 1. There was no statistically significant difference between the two cohorts scanned at different field strengths. Patients in both cohorts had mild global LV systolic impairment with small-to-moderate sized infarcts.

**Table 1:** Patient characteristics

Baseline parameter	1.5 T (n = 10)	3.0 T (n = 10)	p-value
Age, years	56 ± 9	55 ± 12	0.84
Hypertension, n (%)	2 (20)	1 (10)	1.00
Hypercholesterolaemia, n (%)	3 (30)	1 (10)	0.58
Smoking Status, n (%):			0.74
Current	4 (40)	3 (30)	
Ex-smoker	4 (40)	3 (30)	
Never	2 (20)	4 (40)	
Admission glucose, mmol l^−1^	6.6 (5.9–8.9)	6.6 (5.9–7.9)	0.53
BMI, kg m^−2^	28.0 ± 2.4	28.5 ± 4.6	0.77
Anterior STEMI, n (%)	4 (40)	6 (60)	0.66
Time from MI to CMR, days	3.3 (2.3–4.2)	3.4 (2.8–4.6)	0.66
LVEDVI, ml m^−2^	86.8 ± 17.8	90.4 ± 8.07	0.56
LVESVI, ml m^−2^	46.9 ± 13.5	46.9 ± 5.76	0.99
LVEDMI, g m^−2^	67.2 ± 13.0	61.0 ± 8.12	0.22
LV ejection fraction, %	46.4 ± 6.8	48.1 ± 4.2	0.51
Infarct Size, % (of LV mass)	15.8 ± 8.3	11.6 ± 5.2	0.19

*BMI* body mass index, *BP* blood pressure, *CMR* cardiovascular magnetic resonance, *HR* heart rate, *LV* left ventricular, *LVEDMI* left ventricular end-diastolic mass index, *LVEDVI* left ventricular end-diastolic volume index, *LVESVI* left ventricular end-systolic volume index, *MI* myocardial infarction, *STEMI* ST-segment elevation myocardial infarction

### Baseline strain (*Ecc*) and strain rate (PEDSR)

**Fig. 3:**
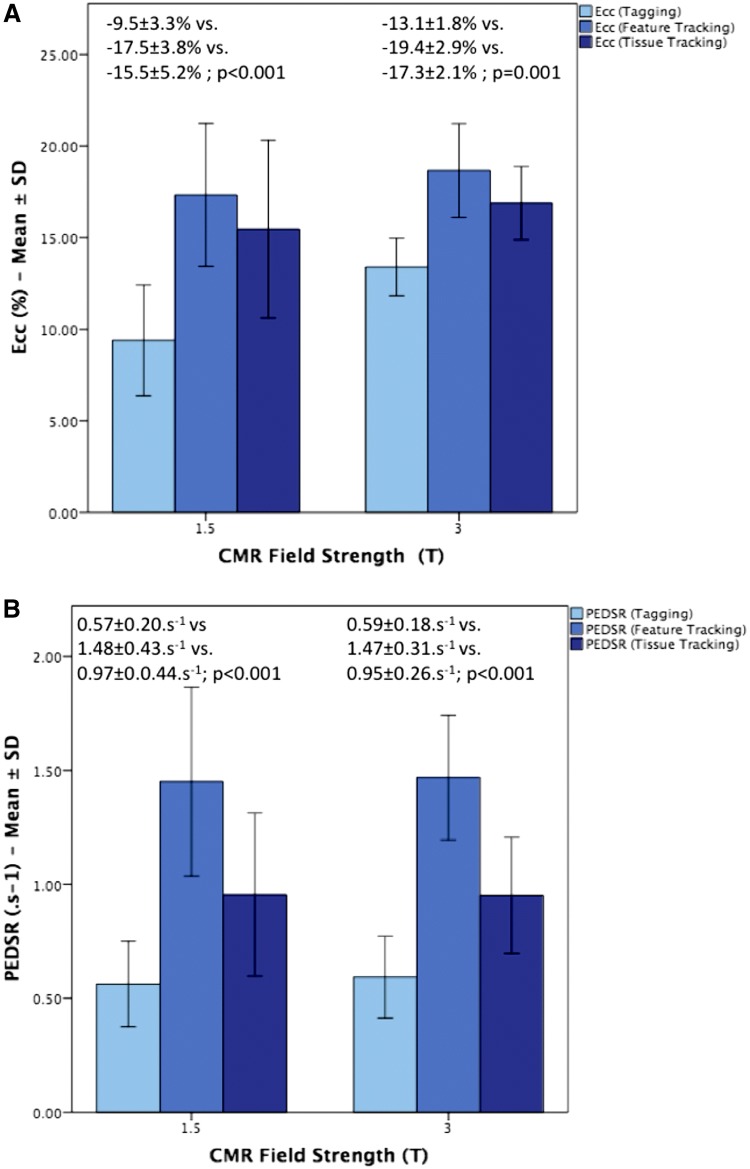
Comparison of *Ecc* and PEDSR by tagging, FT and TT at 1.5 T and 3.0 T CMR using ANOVA of repeated measures. *Ecc* Global Circumferential Strain, *FT* feature tracking, *PEDSR* global circumferential peak early diastolic strain rate, *SD* standard deviation, *TT* tissue tracking. Note: Error bars represent standard deviations (SD)

Results for *Ecc* and PEDSR for the three techniques at both field strengths are shown in Fig. 3. For *Ecc*, tagging produced significantly lower values than FT (mean difference − 8.0%, p < 0.001 at 1.5 T; − 6.3%, p < 0.001 at 3.0 T) and TT (mean difference − 6.0%, p = 0.005 at 1.5 T; − 4.1%, p < 0.001 at 3.0 T). This was also true for PEDSR for tagging versus FT (mean difference − 0.9 s^−1^, p < 0.001 at 1.5 T; − 0.95 s^−1^, p < 0.001 at 3.0 T) and tagging versus TT (mean difference − 0.4.s^−1^, p = 0.01 at 1.5 T; − 0.3 s^−1^, p = 0.01 at 3.0 T). FT produced significantly higher values than TT for both *Ecc* (mean difference 2.0%, p = 0.02 at 1.5 T; 2.2%, p = 0.02 at 3.0 T) and for PEDSR (mean difference 0.5 s^−1^, p = 0.002 at 1.5 T; − 0.7 s^−1^, p < 0.001 at 3.0 T). Agreement between tagging and both FT and TT was poor-to-moderate for *Ecc* and PEDSR at both field strengths, whilst that between FT and TT was slightly better—see Supplemental Table 1.

### Inter-study repeatability

#### Tagging

**Fig. 4:**
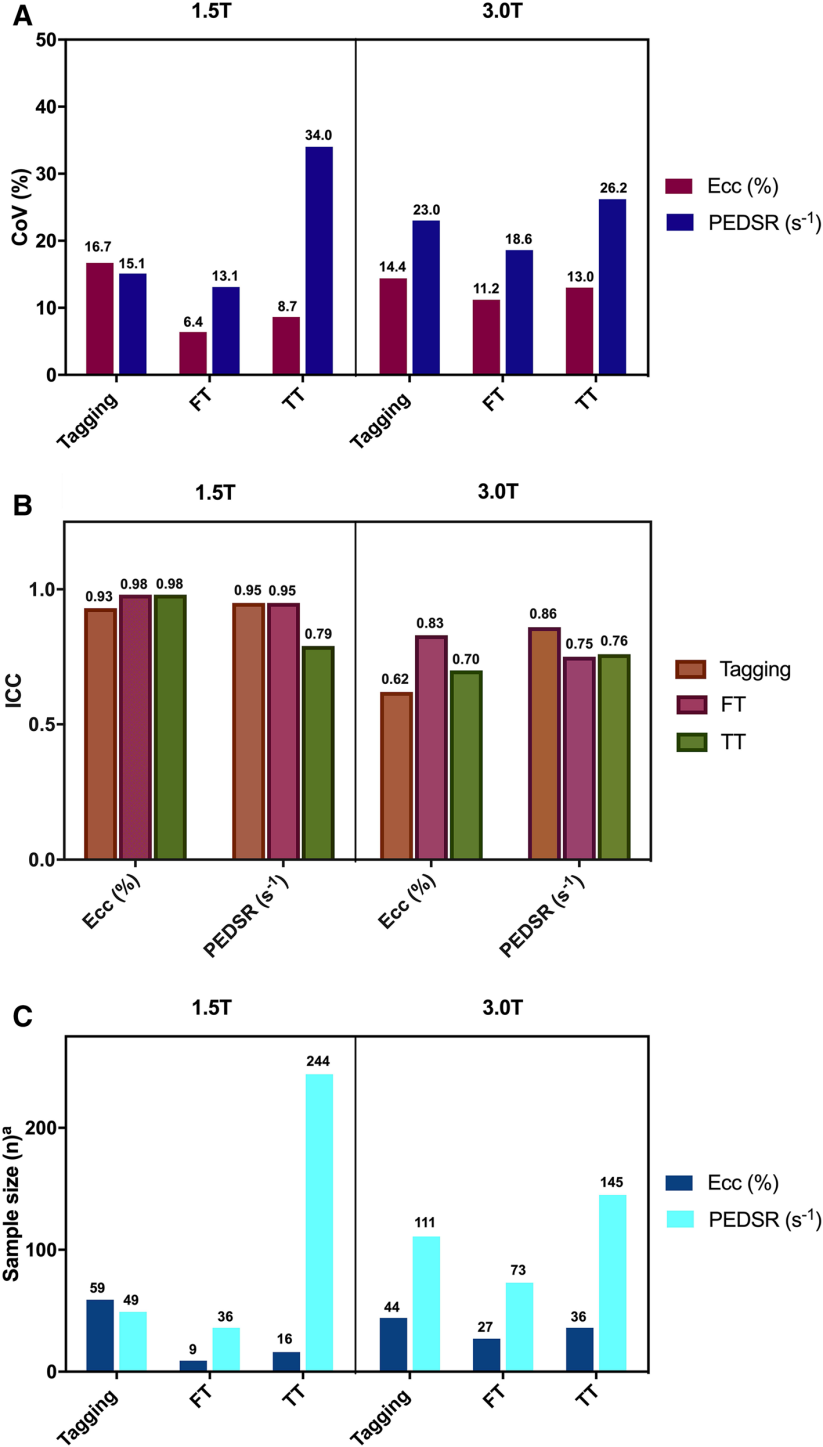
Inter-study repeatability of *Ecc* and PEDSR by tagging, FT and TT at 1.5 T and 3.0 T CMR. Charts showing (**a**) CoV, (**b**) ICC and (**c**) Sample size required to detect a 10% relative change (α = 0.05, Power = 90%). *CMR* cardiovascular magnetic resonance, *CoV* coefficient of variation, *Ecc* global circumferential strain, *FT* feature tracking, *ICC* intra-class correlation coefficient, *PEDSR* global circumferential peak-early diastolic strain rate, *TT* tissue tracking

**Fig. 5:**
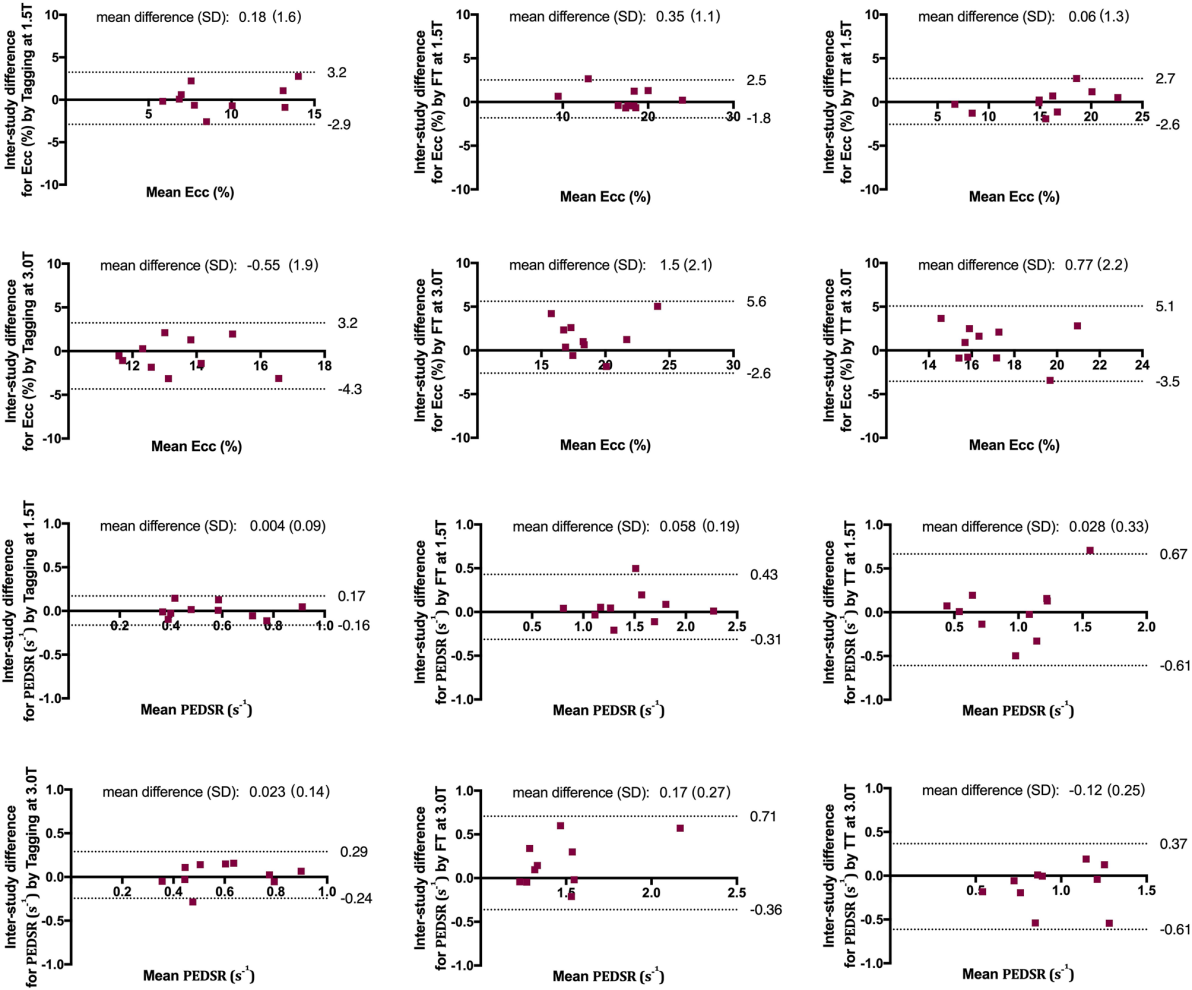
Bland–Altman charts demonstrating the Inter-study differences of *Ecc* and PEDSR by tagging, FT and TT at 1.5 T and 3.0 T CMR. *Ecc* global circumferential strain, *FT* feature tracking, *PEDSR* global circumferential peak-early diastolic strain rate, *TT* tissue tracking

Inter-study repeatability for tagging was good for *Ecc* at both field strengths (CoV of 16.7% at 1.5 T and 14.4% at 3.0 T)—see Fig. 4 and Fig. 5. For PEDSR, repeatability was good at 1.5 T (CoV 15.1%) and moderate at 3.0 T (CoV 23.0%).

#### Feature tracking

*Ecc* by FT had excellent repeatability at 1.5 T (CoV 6.4%) and good repeatability at 3.0 T (CoV 11.2%)—see Fig. 4 and Fig. 5. Repeatability for PEDSR by FT was good at both field strengths (CoV 13.1% at 1.5 T and 18.6% at 3.0 T).

#### Tissue tracking

TT had excellent inter-study repeatability for *Ecc* at 1.5 T (CoV 8.65%) and good repeatability at 3.0 T (CoV 13.0%) – see Fig. 4 and Fig. 5. For PEDSR, repeatability was poor at 1.5 T (CoV 34.0%) and moderate at 3.0 T CMR (CoV 26.2%).

### Intra- and inter-observer variability

#### Tagging

For *Ecc*, tagging had good-to-excellent intra- and inter-observer variability at both field strengths (CoV between 4.0 to 14.6%)—see Table 2 and Supplemental Figs. 1 and 2. For PEDSR, observer variability was good-to-moderate (CoV between 11.0 to 20.3%).

**Table 2:** Intra- and Inter-observer variability of *Ecc* and PEDSR by tagging, FT and TT at 1.5 T and 3.0 T CMR

	Tagging		Feature tracking (FT)		Tissue tracking (TT)	
	Intra-observer	Inter-observer	Intra-observer	Inter-observer	Intra-observer	Inter-observer
1.5 T (n = 10)						
**Ecc (%)**						
COV (%)	9.3	14.6	5.2	6.5	8.4	11.1
ICC	0.98	0.95	0.99	0.98	0.98	0.97
Mean Diff (SD)	0.11 (0.89)	0.56 (1.4)	− 0.27 (0.91)	0.59 (1.1)	0.19 (1.3)	− 0.15 (1.7)
LoA	− 1.64 to + 1.86	− 2.14 to + 3.25	− 2.04 to + 1.51	− 1.58 to + 2.76	− 2.35 to + 2.72	− 3.53 to + 3.24
**PEDSR (s**^**−1**^**)**						
COV (%)	14.8	20.3	10.1	10.3	13.1	13.6
ICC	0.96	0.94	0.97	0.96	0.96	0.95
Mean Diff (SD)	− 0.05 (0.08)	− 0.003 (0.11)	− 0.02 (0.15)	0.06 (0.15)	0.01 (0.12)	− 0.001 (0.13)
LoA	− 0.21 to + 0.11	− 0.21 to + 0.21	− 0.31 to + 0.27	− 0.22 to + 0.35	− 0.22 to + 0.25	− 0.25 to + 0.25
3.0 T (n = 10)						
**Ecc (%)**						
COV (%)	4.0	9.2	4.3	6.5	10.3	4.4
ICC	0.99	0.93	0.95	0.92	0.88	0.97
Mean diff (SD)	0.23 (0.54)	− 0.22 (1.3)	− 0.23 (0.81)	0.73 (1.2)	0.24 (1.8)	0.53 (0.75)
LoA	− 0.83 to + 1.29	− 2.70 to + 2.26	− 1.82 to + 1.36	− 1.60 to + 3.05	− 3.19 to + 3.68	− 0.93 to + 2.00
**PEDSR (s**^**−1**^**)**						
COV (%)	11.0	20.2	7.0	6.0	10.1	20.8
ICC	0.97	0.93	0.95	0.97	0.98	0.90
Mean Diff (SD)	− 0.04 (0.07)	− 0.06 (0.12)	− 0.003 (0.1)	0.02 (0.09)	− 0.05 (0.1)	− 0.001 (0.2)
LoA	− 0.17 to + 0.09	− 0.30 to + 0.18	− 0.21 to + 0.20	− 0.16 to + 0.19	− 0.24 to + 0.14	− 0.39 to + 0.39

*CMR* Cardiovascular magnetic resonance, *CoV* coefficient of variation, *Ecc* global circumferential strain, *FT* feature tracking, *ICC* intra-class correlation coefficient, *LoA* 95% limits of agreement, *PEDSR* global circumferential peak-early diastolic strain rate, *TT* tissue tracking

#### Feature tracking

FT had excellent intra- and inter-observer variability for *Ecc* at both field strengths (CoV between 4.3 to 6.5%)—see Table 2 and Supplemental Figs. 1 and 2. For PEDSR, this was excellent at 1.5 T (CoV ~ 6.0%) and good at 3.0 T (CoV ~ 10.0%).

#### Tissue tracking

For *Ecc*, TT had good-to-excellent observer variability at both field strengths (CoV between 4.4 to 11.1%)—see Table 2 and Supplemental Figs. 1 and 2. For PEDSR, observer variability was good-to-moderate (CoV between 10.1 to 20.8%).

### Correlation with baseline infarct size

The correlation between IS and *Ecc* was moderate and similar when strain was assessed by TT (r = − 0.57, p = 0.01) and FT (r = − 0.55, p = 0.012) for the total cohort (n = 20), and there was a also a moderate correlation for tagging just failing to reach significance(r = − 0.43, p = 0.06). There was no significant correlation between IS and PEDSR assessed by tagging (r = − 0.09, p = 0.70), FT (r = − 0.15, p = 0.54) or TT (r = − 0.08, p = 0.73) for the whole cohort.

### Sample size calculations

Based on the current results, tagging requires considerably larger sample sizes (n = 59) than FT (n = 9) or TT (n = 16) to detect a 10% relative change in *Ecc* at 1.5 T but similar sample sizes are required at 3.0 T (tagging: n = 44; FT: n = 27; TT: n = 36)—see Fig. 4. For PEDSR, sample size requirements were higher than for *Ecc* for all three techniques at both field strengths (except for tagging at 1.5 T). Notably, sample size requirements for detecting a 10% relative change in PEDSR appeared to be lower for FT than tagging or TT at both field strengths.

## Discussion

This is the first study to establish inter-study repeatability of global strain parameters assessed by CMR for three separate strain assessment techniques following STEMI. Furthermore, we have compared these techniques at two clinically used field strengths, 1.5 T and 3.0 T.

### Inter-study repeatability

Inter-study repeatability for *Ecc* by tagging was good whilst it was good-to-excellent for both FT and TT. Inter-study repeatability for PEDSR for FT and TT techniques was lower than that of *Ecc*, and similar for tagging, but was still moderate-to-good at both field strengths (except for TT at 1.5 T, which showed poor repeatability with CoV of 34.0%)—this mirrors the results seen in previous studies with aortic stenosis and STEMI patients [18, 24]. This may be partly attributable to sub-optimal contour tracking in the diastolic phase when differentiation between trabeculae and compact myocardium may be particularly difficult. Our results are consistent with those from a previous study involving patients with aortic stenosis, in which inter-study repeatability of *Ecc* was good for tagging (CoV 13.0–19.0%) and excellent for FT (CoV 9.0–10.0%) and repeatability of PEDSR was moderate overall (CoV 19.0–34.0% for tagging and 14.0–26.0% for FT) [18]. Tagging sequences (especially SPAMM) are susceptible to poor image quality due to prolonged image acquisition time, long breath holding, and diastolic tag fading; this is especially the case with 1.5 T CMR due to decreased T1 relaxation time compared to 3.0 T [19], which may explain why reproducibility of *Ecc* by tagging was better at 3.0 T compared with 1.5 T. Cine SSFP sequences, on the other hand, are more prone to artifacts at 3.0 T due to greater field inhomogeneity resulting in poorer inter-study repeatability of *Ecc* by FT and TT at 3.0 T compared with 1.5 T. However, cine sequences are quick to acquire and result in both high signal and contrast to noise ratio, which is constant throughout the cardiac cycle. Consequently, tagging may be more vulnerable to lower inter-scan repeatability than cine-methods and this may be why inter-study repeatability appeared better for FT and TT in our cohort at 1.5 T.

### Intra- and inter-observer variability

Intra- and inter-observer variability of FT and TT appeared similar to that of tagging, although variability was lower for *Ecc* than for PEDSR. These results are similar to those seen previously in the STEMI population (*Ecc:* CoV of 2.1–6.0% for FT, 13.1–22.2% for tagging) and in patients with aortic stenosis (*Ecc*: CoV of ~ 4.0% for FT, ~ 5.0% for tagging; PEDSR: CoV of ~ 6.0% for both FT and tagging) [18, 24]. These findings are likely to be explained by standardisation in image analysis (i.e. contour definition and re-adjustments) reducing variability within and between readers. Overall, intra- and inter-observer variability for both *Ecc* and PEDSR was slightly better at 3.0 T than at 1.5 T with the three techniques, suggesting that myocardial function may be better defined and more robust throughout the cardiac cycle at 3.0 T, owing to T1 lengthening and superior myocardial signal- and contrast-to-noise ratios compared with 1.5 T [16].

### Inter-technique agreement and correlation

Strain parameters assessed by the three techniques cannot be used interchangeably. As seen previously [18, 24], we found tagging produced significantly lower values for *Ecc* and PEDSR than both FT and TT irrespective of field strength. This may be explained by the fact that, unlike tagging and TT, TomTec’s FT software automatically assigns ‘zero’ strain values to dyskinetic segments resulting in higher (more negative) strain values. Both tagging (via local sine wave modelling) and TT (by following the motion of myocardial nodes on SSFP cines) evaluate myocardial motion between the user-defined endocardium and epicardium, hence providing a transmural assessment of strain parameters [21, 33]. By contrast, FT calculates strain by separately tracking motion five pixels perpendicular to the endocardial and epicardial borders through the cardiac cycle [20]. It therefore is likely to give an overestimation of *Ecc*/PEDSR in areas of overlap around the mid-myocardium when endocardial and epicardial values are averaged. This may also explain why *Ecc*/PEDSR appeared to be significantly higher with FT than with both tagging and TT. However, agreement between FT and TT (both of which use the same sequences for analysis) was good-to-excellent whilst that between tagging and FT, and tagging and TT, was poor, suggesting that inherent inter-sequence differences may have a greater contribution to the lack of accord between the three techniques than disparities between their software algorithms.

We found that there was moderate correlation between *Ecc* and IS for tagging (r = 0.43, p = 0.06), FT (r = − 0.55, p = 0.01) and TT (r = − 0.57, p = 0.01) and these results are similar to those seen previously using FT in STEMI (r = − 0.40, p = 0.06) [24]. However, there was no correlation between PEDSR and IS by any technique. Whilst diastolic dysfunction may be an important predictor of outcome post-infarction as seen previously [2], given that patients in our study tended to have small- medium sized infarcts, other factors such as age, hypertension and LV mass are likely to have a more dominant effect in this relatively small population. Further work evaluating PEDSR post-STEMI with significantly larger sample sizes may be useful.

### Determination of myocardial strain at 1.5 T versus 3.0 T

We observed generally lower strain values at 1.5 T compared with 3.0 T in our study. Previous studies that have assessed strain at both CMR field strengths have also yielded slightly lower values with 1.5 T compared with 3.0 T [14, 18]. These studies were performed on healthy volunteers and patients with aortic stenosis, respectively, and therefore the effect of an infarct alone would not explain this discrepancy. One possible explanation is that the higher spatial resolution and signal-to-noise ratio achieved with scanning at 3.0 T compared with 1.5 T may allow superior *tracking* of myocardial deformation (and thus result in slightly *higher*, and possibly more *accurate*, strain values). Nonetheless, the observed differences in strain values were small between field strengths and, in the study conducted by Schuster et al., shown not to be statistically significant. The exact reason for the observed discrepancy between strain values determined at the two CMR field strengths remains unclear, however, in all studies (including ours), different patients were recruited to undergo a scan at 1.5 T versus 3.0 T and hence patient differences may be a potential contributing factor; It would be interesting to ascertain whether this difference persists if the same patients undergo a scan on both 1.5 T and 3.0 T CMR platforms and this is perhaps an area for future study.

### Optimal method to assess strain on CMR post-STEMI

Tagging-based methods to analyse strain on CMR have been considered the gold-standard and SPAMM-tagging is said to be the only technique to be validated in-vivo with sonomicrometry [39]. This validation study had a number of limitations including (1) small sample size, (2) imperfect matching of SPAMM and sonomicrometry measurement sites, (3) differing timing of data acquisition and (4) wide 95% limits of agreement. We believe that the *accuracy* of strain assessed (i.e. how close the calculated strain value is to the *true* value of strain) may not be as relevant to validation of the three techniques as is the *precision* (i.e. in terms of observer variability and test–retest repeatability) of the strain being measured. We have shown that tagging appears to have lower inter-study repeatability than FT and TT. This suggests that cine-based CMR techniques to assess strain are likely to be more useful than tagging for monitoring response to treatment and progression of LV dysfunction.

Tagging is also associated with increased scan time and laborious post-processing analysis (especially for PEDSR), as highlighted in previous studies [13, 18, 24]. Furthermore, our data suggest that tagging requires a considerably larger sample size to detect a 10% relative change in *Ecc* compared to FT or TT at 1.5 T CMR that may hamper its utility in interventional studies. Strain assessment by FT or TT would reduce scan time by obviating the need to acquire additional sequences (as needed with tagging). This makes both FT and TT attractive alternatives to tagging for CMR-based strain assessment. Both techniques have similar observer variability and inter-study repeatability although FT appears more reliable overall at both 1.5 T and 3.0 T with consistently lower sample size requirements. However, TT has the advantage that volumetric analysis is routinely performed on SSFP-cine SAX slices, which can then be *imported* to the TT module on *cmr42* software to compute strain. This allows volumetric, functional and strain analysis to be performed on a single software platform without requiring further contours to be drawn specifically for strain assessment (as required with FT). CMR-TT is therefore a novel, robust (reproducible) and practical (time-economical) tool to assess strain on CMR post-STEMI at both 1.5 T and 3.0 T.

### Limitations

The main limitation of this study is the small sample size. However, this is comparable with previously published studies reporting inter-study repeatability of strain assessment on CMR [14–16, 18, 23, 40]. Different patients were scanned at the different field strengths and this may have impacted on the results although we mitigated this with the randomized design and there were no major significant differences between the groups. Ours is a single-centre and single CMR vendor study utilising a single tagging sequence; hence these results may not be generalizable to other tagging sequences (although *Ecc* measured by Intag and Harmonic Phase analysis [HARP] have shown good agreement previously [33]). Using a CSPAMM tagging sequence may improve tag persistence [7], but we used SPAMM-tagging given its wider availability, which lends itself to multi-centre collaboration for strain assessment in clinical trials. We did not acquire long axis images using the tagging sequence and we were not therefore able to compare GLS by tagging with that by FT and TT due to concerns that acquisition of additional (long axis tagging) sequences would increase total scan time and place additional breath-holding burden on patients (particularly across two scans) acutely following their recent STEMI. We have therefore only reported peak circumferential strain (*Ecc*) in this study as this has consistently been shown to be the most reproducible strain parameter with the least inter-technique (vendor) variation; it is therefore likely to be the most sensitive strain parameter to detect inter-study variation [13–16]. Furthermore, we only recruited males to limit gender bias, given our small sample size, meaning our results may not translate cross-gender although CMR-*Ecc* has been shown to have the least variation and no significant gender difference when measured by both FT and tagging [17]. We only assessed the repeatability of *global* strain and not segmental strain as we have previously shown that the latter has high intra- and inter-observer variability (CoV between 26 to 60%) for both tagging and FT [24]. Scanning patients twice on the same day may carry the limitation of less patient compliance on the second scan and thus potentially more breathing artefacts (due to fatigue) and reduced image quality, which could bias inter-study repeatability results. However, the tagging and SSFP cine images were acquired in random order for the first scan and then in reverse order for the second scan to mitigate bias. Finally, our results cannot be extrapolated to the ‘normal’ population and they are only applicable in the setting of STEMI as the studied cohort and using the method that we applied (selection of identical SAX slices for all three strain analysis methods), particularly given that inter-study variability of PEDSR has recently been shown to be very good using TT when the entire SAX stack is analysed to compute strain [25].

## Conclusions

Following STEMI, *Ecc* and PEDSR are higher when measured with FT and TT than with tagging. Inter-study repeatability of *Ecc* is good for tagging, excellent for FT and TT at 1.5 T, and good for all three methods at 3.0 T. CMR. The repeatability of PEDSR is good to moderate at 1.5 T and moderate at 3.0 T. Cine-based methods to assess *Ecc* following STEMI may be preferable to tagging.

## Electronic supplementary material

Below is the link to the electronic supplementary material.

Supplementary file 1 (DOCX 33 kb)

Supplementary file 2 (TIFF 2787 kb)—Supplemental Figure 1: Bland-Altman charts demonstrating the Intra-observer differences of Ecc and PEDSRby tagging, FT and TT at 1.5T and 3.0T CMR. Ecc, Global Circumferential Strain; FT, Feature Tracking; PEDSR, Global Circumferential Peak-Early Diastolic Strain Rate; TT, Tissue Tracking

Supplementary file 3 (TIFF 2801 kb)—Bland-Altman charts demonstrating the Inter-observer differences of Ecc and PEDSRby tagging, FT and TT at 1.5T and 3.0T CMR. Ecc, Global Circumferential Strain; FT, Feature Tracking; PEDSR, Global Circumferential Peak-Early Diastolic Strain Rate; TT, Tissue Tracking

## Abbreviations

CMR: Cardiovascular magnetic resonance imaging
CoV: Coefficient of variation
Ecc: Global peak circumferential systolic strain
FT: Feature tracking
IS: Infarct size
LV: Left ventricular
PEDSR: Peak early diastolic strain rate
SAX: Short axis
SPAMM: Spatial modulation of magnetisation
SSFP: Steady-state free-precession
STEMI: ST-segment elevation myocardial infarction
TT: Tissue tracking
