# Supplementary material for: Inter-study repeatability of circumferential strain and diastolic strain rate by CMR tagging, feature tracking and tissue tracking in ST-segment elevation myocardial infarction
Source: Int J Cardiovasc Imaging. 2020 Mar 9;36(6):1133–46. doi: 10.1007/s10554-020-01806-8 (PMC7228913; doi:10.1007/s10554-020-01806-8)
Supplement: Supplementary file 1 — (DOCX 33 kb) [file 10554_2020_1806_MOESM1_ESM.docx]

Supplemental Figure 1: Bland-Altman charts demonstrating the Intra-observer differences of *Ecc* and PEDSR by tagging, FT and TT at 1.5T and 3.0T CMR

**Abbreviations:** Ecc, Global Circumferential Strain; FT, Feature Tracking; PEDSR, Global Circumferential Peak-Early Diastolic Strain Rate; TT, Tissue Tracking

Supplemental Figure 2: Bland-Altman charts demonstrating the Inter-observer differences of *Ecc* and PEDSR by tagging, FT and TT at 1.5T and 3.0T CMR

**Abbreviations:** Ecc, Global Circumferential Strain; FT, Feature Tracking; PEDSR, Global Circumferential Peak-Early Diastolic Strain Rate; TT, Tissue Tracking

Supplemental Table 1: Inter-technique correlation and agreement of Ecc and PEDSR measured by Tagging, FT and TT on 1.5T and 3.0T CMR

|  | **Mean Difference**  **(p-value)** | **Correlation (*r*)**  **(p-value)** | **ICC** | **CoV (%)** |
| --- | --- | --- | --- | --- |
| **1.5T** | | | | |
| ***Ecc* (%)** |  |  |  |  |
| **Tagging vs FT** | -8.03 (p<0.001) | 0.14 (p=0.69) | 0.25 | 34.4 |
| **Tagging vs TT** | -6.02 (p=0.005) | 0.31 (p=0.38) | 0.44 | 41.8 |
| **FT vs TT** | 2.01 (p=0.024) | 0.91 (p<0.001) | 0.93 | 14.3 |
| **PEDSR (s^-1^)** |  |  |  |  |
| **Tagging vs FT** | -0.92 (p<0.001) | 0.56 (p=0.09) | 0.58 | 35.4 |
| **Tagging vs TT** | -0.40 (p=0.007) | 0.55 (p=0.099) | 0.57 | 48.5 |
| **FT vs TT** | 0.51 (p=0.002) | 0.61 (p=0.062) | 0.76 | 31.5 |
| **3.0T** | | | | |
| ***Ecc* (%)** |  |  |  |  |
| **Tagging vs FT** | -6.29 (p<0.001) | 0.47 (p=0.171) | 0.59 | 16.0 |
| **Tagging vs TT** | -4.14 (p<0.001) | 0.27 (p=0.449) | 0.42 | 15.3 |
| **FT vs TT** | 2.15 (p=0.021) | 0.57 (p=0.086) | 0.70 | 13.3 |
| **PEDSR (s^-1^)** |  |  |  |  |
| **Tagging vs FT** | -0.95 (p<0.001) | 0.60 (p=0.07) | 0.68 | 26.9 |
| **Tagging vs TT** | -0.29 (p=0.005) | 0.59 (p=0.073) | 0.71 | 32.3 |
| **FT vs TT** | 0.67 (p<0.001) | 0.45 (p=0.195) | 0.61 | 28.6 |
| **Abbreviations**: Ecc, Global Circumferential Strain; CoV, Coefficient of Variation; ICC, Intra-class correlation coefficient; PEDSR, Global Circumferential Peak-Early Diastolic Strain Rate | | | | |
