# Supplementary figures and images for: Inter-study repeatability of circumferential strain and diastolic strain rate by CMR tagging, feature tracking and tissue tracking in ST-segment elevation myocardial infarction
Source: Int J Cardiovasc Imaging. 2020 Mar 9;36(6):1133–46. doi: 10.1007/s10554-020-01806-8 (PMC7228913; doi:10.1007/s10554-020-01806-8)

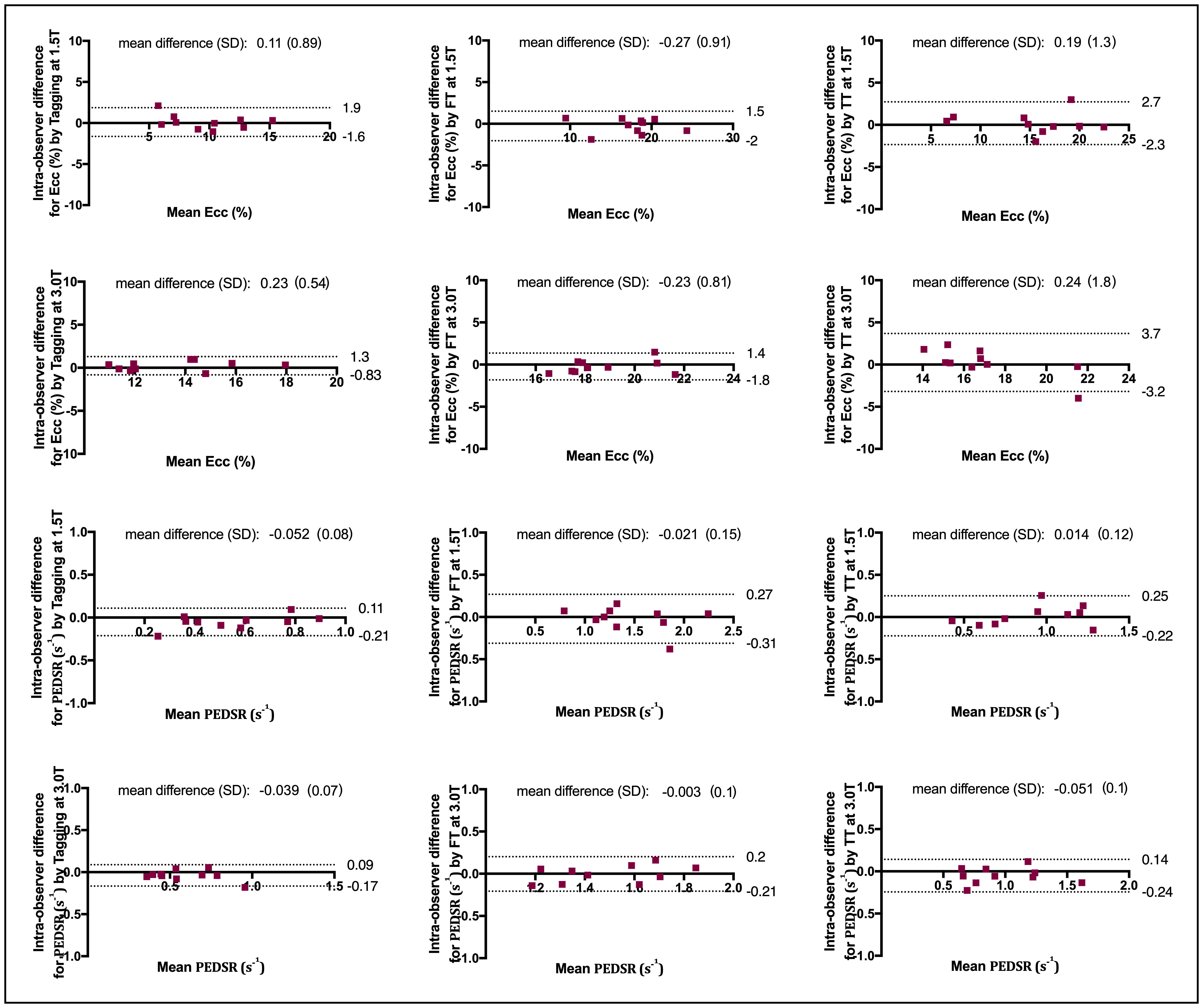

Supplement: Supplementary file 2 — (TIFF 2787 kb)—Supplemental Figure 1: Bland-Altman charts demonstrating the Intra-observer differences of Ecc and PEDSRby tagging, FT and TT at 1.5T and 3.0T CMR. Ecc, Global Circumferential Strain; FT, Feature Tracking; PEDSR, Global Circumferential Peak-Early Diastolic Strain Rate; TT, Tissue Tracking [file 10554_2020_1806_MOESM2_ESM.tiff]

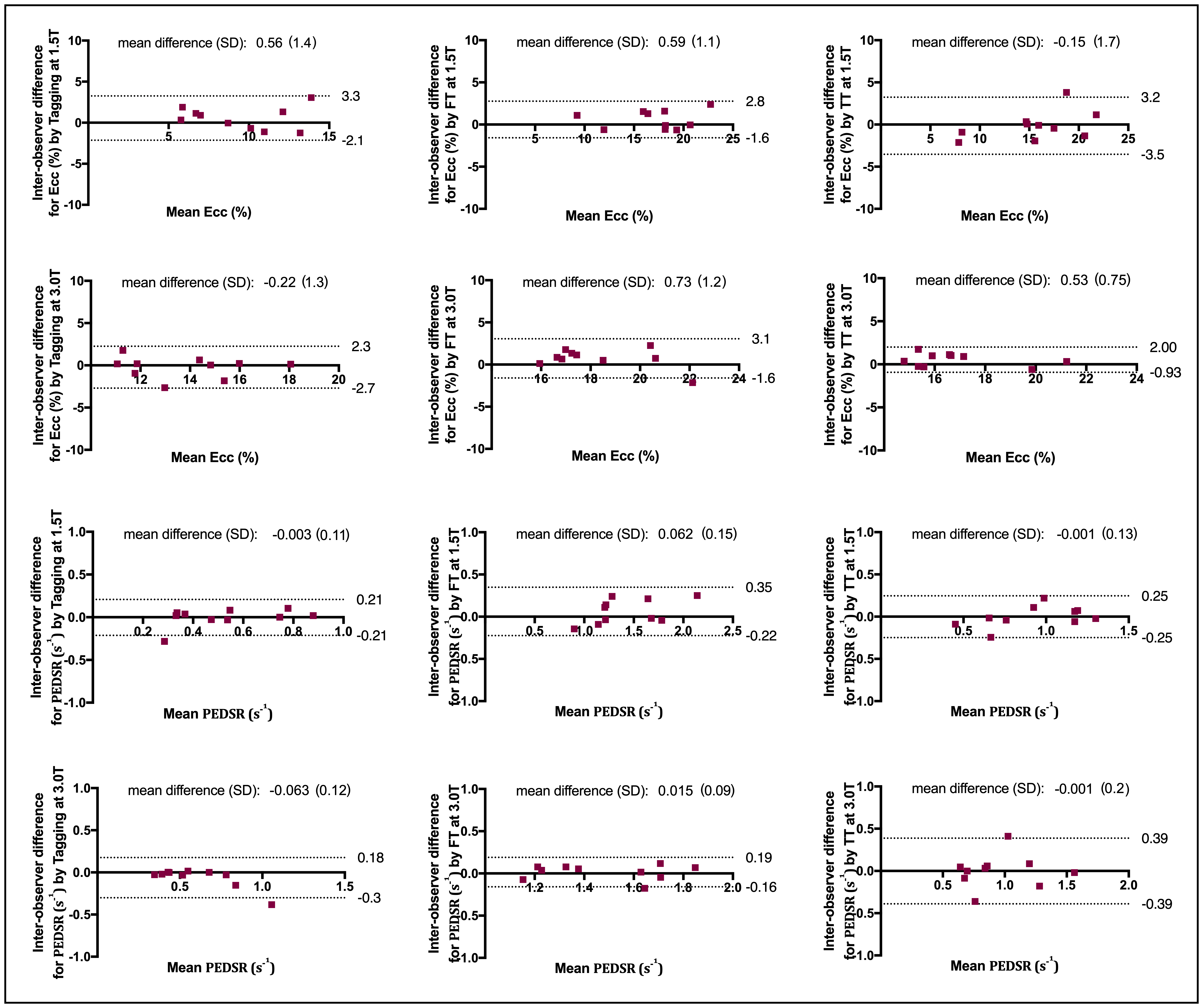

Supplement: Supplementary file 3 — (TIFF 2801 kb)—Bland-Altman charts demonstrating the Inter-observer differences of Ecc and PEDSRby tagging, FT and TT at 1.5T and 3.0T CMR. Ecc, Global Circumferential Strain; FT, Feature Tracking; PEDSR, Global Circumferential Peak-Early Diastolic Strain Rate; TT, Tissue Tracking [file 10554_2020_1806_MOESM3_ESM.tiff]
